# A review on peripheral blood CD4+ T lymphocyte counts in healthy adult Indians

**Published:** 2010-12

**Authors:** Ashwini Shete, Madhuri Thakar, Philip Raj Abraham, Ramesh Paranjape

**Affiliations:** National AIDS Research Institute (ICMR), Pune, India

**Keywords:** CD4, HIV, Reference range, T lymphocytes

## Abstract

The CD4+ T lymphocytes are the crucial cells in the cascade of events in forming immune response to the foreign antigen and hence monitoring the CD4+ T cell counts to understand the extent of immune deficiency is a common practice. CD4+ T cells are also the primary target cells for human immunodeficiency virus (HIV). Hence CD4+ T lymphocyte count is the most important marker of immune dysfunction in HIV disease progression. The estimation of CD4+ T cell counts is used to decide the initiation of anti retroviral therapy (ART), to monitor the efficacy of ART and to start treatment for opportunistic infections (OIs). To develop the threshold levels of CD4+ T cell counts, data from western countries are being used in India. The CD4+ T cell counts are known to be influenced by race and environmental factors. Hence it is important to establish the reference ranges for the CD4+ T cell counts in the target population to understand the immune dysfunction. The information on the lower limits of the CD4+ T cells count is necessary to decide the initiation and monitoring of ART. The published data on the CD4+ T cells count in healthy Indian adult population have been reviewed, analyzed and discussed in this review article. The requirement of establishment of reference ranges in Indian population is discussed.

CD4+ T helper lymphocytes play a central role in regulation of immune response^1^. They have capacity to help B cells for generating antibodies, to recruit and activate macrophages, to recruit neutrophils, eosinophils, and basophils to sites of infection and inflammation^2^. The immunophenotyping of the lymphocytes, especially CD4+ T cells from peripheral blood is being used to assess the extent of immune dysfunction in the primary and secondary immunodeficiency, chronic infectious diseases and various cancers such as Hodgkin’s disease, lymphoma, etc. The clinical applications of immunophenotyping of CD4+ T cells include the monitoring of disease progression in HIV infection^3^,^4^, diagnosis of immunodeficiency disorders^5^,^6^, evaluation of immune-mediated diseases^7^, and the assessment of immune reconstitution following stem cell transplantation^8^.

Decreased CD3+ and CD4+ T cells and increased IgE levels were more frequently observed in the Hodgkin’s disease survivors with recurrent infections. Hence, examination of lymphocyte subpopulations may be helpful in the prediction of an increased risk of recurrent infections in Hodgkin’s disease survivors^9^. The same investigators further reported similar findings in nephroblastoma long term survivors at five years post anti-cancer therapy^10^. The CD4+ T lymphocytes were decreased in disease survivors. Similarly, the depletion of CD4+ T cell in renal transplant recipients is associated with the development of solid cancers and lymphoma^11^. The CD4+ T cell count estimation became an important parameter for monitoring immune deficiency after the discovery of the acquired immune deficiency syndrome (AIDS). One of the main factors that led to defining the AIDS as new clinical entity was the facility available to measure the ratio of helper (CD4+) to suppressor (CD8+) T lymphocytes. All AIDS patients were found to have highly inverted ratio of CD4+ to CD8+ T lymphocytes indicating marked immunosuppression. Subsequently it became evident that HIV infects CD4+ T lymphocytes selectively and causes the destruction of CD4+ T cells directly as well as indirectly leading to gradual loss of the CD4+ T cell numbers in peripheral circulation. Hence the monitoring of the CD4+ T cell count became an important parameter for assessing HIV disease progression^12^,^13^.

## Estimation of CD4+ T cell counts in the peripheral blood

Flowcytometry is the gold standard for the estimation of CD4+ T cell counts due to its accuracy, precision and reproducibility and hence is used widely. This technology is capable of high sample throughput and offers great versatility in its applications. However, flow cytometry based CD4+ T cell estimation is relatively complex, and technically demanding. The equipment is costly and needs regular maintenance. Additionally, it is essential that operators of the flowcytometer be sufficiently trained in the technical and biological aspects of CD4+ T cells measurement. The introduction of simpler and portable instruments working on the modified flowcytometry principles are now available and are being increasingly used throughout the world.

Flowcytometry can be used to estimate CD4+ T cell count using either dual platform or single platform approach. The dual platform flowcytometry approach uses haematology analyzer to measure absolute lymphocyte count and flow cytometer to estimate the relative percentages of CD4+ T cells; the absolute CD4+ T cell number is calculated by multiplying absolute lymphocyte number with percentage of CD4+ cells and dividing the product by 100. The variations in absolute lymphocyte count add to the variations in the output of flowcytometer^14^,^15^. In the single platform approach, the flowcytometer is equipped to give both relative percentages and absolute counts of lymphocyte subsets using single observation eliminating the variations introduced due to haematology analyzer. Hence a single platform approach is now being used widely for estimation of absolute CD4+ T cell count. To date, single-platform technologies have two options, microbead-based technologies and the volumetric technologies^3^. There are a number of alternative technologies for CD4+ T cells estimation reported in literature; however, the flowcytometry is still the method of choice.

## Factors influencing CD4+ T cell counts

The CD4+ T cell count has been shown to be influenced by sex, age, race, time of specimen collection (diurnal rhythms), physical and psychological stress, pregnancy, drug administration (zidovudine, cephalosporin, cancer chemotherapy, nicotine and steroids), tuberculosis, viral infections, presence of anti-lymphocyte auto antibodies and procedures like splenectomy^16^,^17^. Females tend to have higher CD4+ T cell counts than males; on the contrary males have higher CD8+ T cell counts than females^16^,^18^–^20^. Although in adults, age does not have influence on CD4+ T cell counts significantly^20^, decrease in CD4+ T cell counts may be observed in geriatric population.

Apart from physiologic or pathologic conditions, factors that cause variations in the CD4+ T cell counts include instrument used, time of collection and methodologies used for collection, processing and analyzing the whole blood samples. The factors such as integrity of the blood sample, staining reagents and fluorochromes, equipment calibration and performance, gating strategies used for the analysis also add to the variation in the CD4+ T cell counts^21^–^25^. Experience and proficiency in gating and use of lyse-no-wash procedure markedly controlled the inter-laboratory variation in CD4+ T cell counts^26^. The standardized procedures are now also available in terms of CDC/WHO guidelines^27^,^28^ ; strict adherence to guidelines can reduce the variation further.

Even under strict quality control regime, the variations in CD4+ T cell counts measured using different equipment and following different methodologies can be as high as 20 per cent in controlled conditions with adequate technical expertise. Diurnal variation (the variation in the CD4+ T cell counts in the sample collected at different time points during 24 h period in the same individual) adds to this variation by as much as 20 per cent. Malone *et al*^29^ studied the diurnal variation in CD4 counts in HIV-positive and HIV-negative individuals. While both groups reported significant diurnal variation, the variation was greater in HIV-negative individuals. The lowest level of CD4+ T cell counts was seen in the morning around 1000 h, and the highest at around 2000 h. A recent study in the Caribbean population confirmed that there was a progressive increase in CD4+ T cell count throughout the day. It showed that diurnal rhythms influence the lymphocyte subsets in a normal population^30^. Large diurnal variations in CD4+ T cell count and T cell function were observed among HIV infected and non-infected drug users also^31^. Hence for the sake of comparability of values it is recommended that the blood for CD4+ T cell counts should always be drawn at the same time of the day in a given individual.

CD4+ T cells are also known to fluctuate with strenuous physical activity. A study conducted in 20 healthy HIV-negative individuals showed significant decrease in the CD4 lymphocyte count after the rest for 60 min. The baseline mean CD4+ T cell count in these individuals at 0 min was 1060×10^6^ /l which was found to be decreased to 660× 10^6^/l after rest for 60 min (*P* =0.0017)^32^.

## CD4 per cent and CD8 per cent in healthy adult population

As against absolute CD4+ T cell count, CD4 per cent is found to be more stable with respect to time of the day, reagents, equipment used, gating strategies and biological factors that influence the CD4 counts. Though CD4 per cent is not considered a good predictor of HIV disease progression, it indicates whether the rise or drop in CD4+ T cell count is a real change or just a fluctuation. It has been observed that patients with relatively high absolute CD4+ T cells count but low CD4+ T cell percentages experienced faster disease progression than subjects with low CD4+ T cell counts but high CD4 percentages^33^,^34^. Hence, CD4+ T cell percentage may be used as an additional indicator for monitoring the success of antiretroviral therapy (ART). CD4+ T cell percentage is less variable than absolute count; within-subject coefficient of variation is 18 per cent for CD4 per cent vs. 25 per cent for CD4 count^35^. An assessment of the effects of instrumentation, monoclonal antibody and fluorochrome on flow- cytometric immunophenotyping has suggested that by controlling these parameters the inter-laboratory agreement on CD4+ T cell percentages will greatly improve^21^.

It has been observed that CD4 per cent or CD8 per cent values are also less variable in pregnant women as compared to absolute counts. The absolute CD4+ or CD8 T cell counts are significantly lower in pregnant women as compared to non pregnant women^36^. This could be because of haemodilution which occurs in pregnancy and hence percentages would be more useful indicator of immune function in pregnant women also.

## CD4+ T cells in healthy children

The CD4+ T cell counts are normally higher in children compared to adult population. With increasing age the CD4+ T cell counts decrease to attain adult levels at about three to six years of age. A study conducted by Kotylo *et al*^37^ has shown that the relative and absolute numbers of CD4+ T cells are high at birth, decrease during early childhood, and closely approximate adult reference values after the age of 3 yr. It was shown that the CD4+ T cell count declined with age until the age of 18 yr in Ugandan and Turkish population and until the age of 10 yr in children from Kenya^38^–^40^. The reference ranges for both CD4+ T cell percentages and absolute counts in African children differ from those reported in Europe and North America^38^,^41^,^42^. These age related variations in absolute CD4+ T cell counts in children present a challenge while monitoring disease in HIV infected children.

The studies conducted in paediatric population have shown relatively stable CD4 per cent in different age groups in children and hence, usually CD4+ T cell percentages are referred for monitoring children with HIV infection and management of ART^43^–^46^.

## Need for estimation of reference ranges for CD4+ T cells

The information on the reference ranges of CD4+ T cell counts in a population is required for the application in the clinical settings such as various immune deficiencies. Reference values help in proper assessment of the degree of immunodeficiency in various conditions including HIV infection. The lower normal limits of CD4+ T cell counts are important in the routine diagnosis for interpreting putative HIV-associated changes^47^,^48^.

## Reference ranges for CD4 counts in different geographic location

Studies have been carried out worldwide to establish reference ranges for CD4+ T cell counts. Variations in the reference ranges for CD4+ T cell counts have been observed in different populations. In United Kingdom, the mean CD4+ T cell count in normal healthy persons was 830 ± 290 cells/µl^47^. The mean CD4+ T cell counts varied from 868 to 1036 cells/µl in healthy adult non-smoker Caucasian populations in different studies in Western populations^19^,^49^. Mean/median CD4+ T cell counts reported from other parts of world also fall approximately in the same range such as 910±310 cells/µl for Thai population^50^, 863.9±234.8 for African populations^51^, 727 ± 255 cells/µl for Chinese population and 869±310 for Saudi men^52^,^53^. A study conducted on 232 healthy Asian individuals reported a wide range of CD4 cell counts, (401 to 1451 cells/µl) with a mean and median of 838 and 814 cells/µl respectively^54^. Earlier, a Study conducted in China reported a reference range of 330 to 1508 cells/µl from 208 healthy volunteers (mean 785 and median 730 cells/µl)^55^. Reference range of 559 to 2333 cells/µl (mean=1256 cells/µl) for absolute CD4+ T cell counts was reported in healthy Ugandans and 547 to 1327 (mean= 828 cells/µl) in Nigerian adults^42^,^56^. Similarly, the CD4+ T cell reference range in healthy adults from Turkey was from 437 to 2072 (mean 1095 and median 1055 cells/µl)^57^. Comparison of mean CD4+ T cell counts between Ethiopian and Dutch populations showed considerably lower CD4 T cell counts in Ethiopian population (mean=775 cells/µl) as compared to Dutch populations (mean= 993 cells/µl)^58^. The CD4+ T cell reference range in 102 study participants from Tanzania was reported as 312 to 1368 cells/µl (mean 746 and median 723 cells/µl)^59^. Reference ranges of CD4+ T cell counts in HIV negative blood donors of Botswana was found to be 171-1652 cells/µl (Mean 759 and median 726 cells/µl)^60^. Such a varied difference in CD4 ranges reported in various studies could be because of racial and ethnic differences in the populations studied.

## CD4+ T cell counts in healthy adult Indian population

The literature search revealed 15 studies that have determined CD4+ T cell counts in healthy HIV negative Indian adults, either for determination of reference ranges or as a control population in studies involving patient populations (Table). These studies were conducted in different geographic locations in India. Nine of 15 studies were carried out with the objective of estimating normal range for CD4 count; in four studies values were obtained from normal healthy subjects as a control group in comparison with others. Two studies were carried out with the objective of comparing Indian CD4+ T cell counts with Western population^66^,^67^.

**Table.** CD4+ T cell Reference ranges reported by Indian studies

Geographical location	No. of subjects	Absolute CD4 count (cells/µl)		CD4%		Reference no.
			
		Range	Mean and/ or median	Range	Mean and/ or median	
East (E)	14	-	848/-	-	36/ -	70
	44	Male: 379-1128, Female: 547-1181	Male: 711 /651 Female: 766 /745	-	-	71
West (W)	94	430-1740	865/-	30.75-49.60	40.2/ -	20
	30	539-1627	965/ -	48-68	55.27/ -	36
	252	Male: 374-1398 Female: 380-1493	Male: 727 /705 Female: 845/839	Male: 24.2-55.1 Female: 27.5-65	Male: 36.89/36.60 Female: 41.38/41.60	69
	65	Male: 379-1800 Female: 321-1265	Male: 743.4/690 Female: 790.4/741	-	-	73
North (N)	84	Male: 365-1328 Female: 415-1257	Male:763.6/- Female:797.9/-	-	-	18
	200	304-1864	- / 666	17.5 to 50.6	- /35	64
	40	-	818.4/ -	-	-	66
	125	Male: 640-734 Female: 656-824	Male: 687/ - Female: 740/ -	-	-	65
South (S)	44	-	1048/ -	-	-	76
	99	753.3-844.7	799/ -	-	33.0/ -	67
	30	-	834.6/ -	-	-	68
	213	Male: 383-1347 Female: 448-1593	Male: 865/845, Female: 1021/954	-	40.2/40.1	63
Multicentric study*	1027	-	-	E:----	E-----	61
				W: 15-65	W:39.46/38.75	
				N: 15-60	N: 37.38/37.26	
				S: 14-51	S:32.43/33.0	
						

* The only multicentric study conducted by ICMR Task Force in 1998. The mean and range of CD4 per cent given in the Table is the collective data from 3 centers (north), 2 centers (west) and one center from south

The sample size in 14 of these 15 studies varied from 14 to 213. The only multicentric study was conducted by the National Task Force, constituted by the Indian Council of Medical Research (ICMR)^61^. This study was conducted at six centers *viz*; Chandigarh, Delhi (two centers), Mumbai, Pune and Vellore and the sample size was 1027. However, this study had a major limitation in that the study established reference values only for CD4+ T cell percentages and not absolute CD4 counts.

Majority of the studies have used flowcytometric assays based on either single or dual platform techniques, which applied equipment like FACSSort, FACSCount, FACSCalibur, and Coulter Epics-XL. Two studies used ELISA based assays; Capcellia assays and magnetic bead based microscopic assay respectively^62^,^63^. Although sample collection was restricted to morning hours in a few studies, diurnal variations were not controlled in other studies.

The reported lowest value of CD4+ T cells count varied between 304 to 500 cells/µl and highest CD4+ T cells count varied between 1000 cells/µl to as high as 1864 cells/µl^64^,^65^ indicating the wide difference that exists in the CD4 reference ranges. The mean CD4+ T cell counts as reported in different Indian studies are illustrated in the Figure. The mean CD4+ T cell counts in the north India varied from as low as 703 to 818 cells/µl^65^,^66^, in south India from 799 to 1048 cells/µl^62^,^67^,^68^,^76^, 743 to 965 cells/µl^20^,^69^ in western India and 848 cells/µl^70^ in eastern India. Although CD4+ T cell counts in south Indian population appear higher than those in north in several studies (Fig.), the CD4+ T cell percentages as reported in the only multicentric study were significantly lower in south Indian population compared with the populations in other parts of the country^61^. The females have been observed to have higher CD4+ T cell counts and males have higher CD8+ T cell counts in different Indian studies^18^,^63^,^71^. The mean CD4 per cent in different geographical regions of India varied from 32.43 per cent (south Indian population; range 14 to 51%) to 41.15 per cent (population from western India; range 15 to 65%)^36^,^61^,^64^. Overall, mean CD4 per cent has been found to be lower (37.1 ± 7.8) in the Indian multicentre study as compared to 43.0 per cent ±7.5 reported in US population^48^,^61^. It has been suggested by Lifson *et al*^72^ that a ratio below 0.85 should raise the suspicion of high risk behaviour of acquiring HIV. However, the studies conducted in normal Indian populations showed variations in CD4:CD8 ratio from 0.04 to 3.5^64^. The mean CD4: CD8 ratio varied between 0.94 to 1.78 in different studies^67^,^73^. The ICMR multicentric study has reported a mean CD4:CD8 ratio of 1.2 from the six centers in different locations in India^61^.

**Fig.**
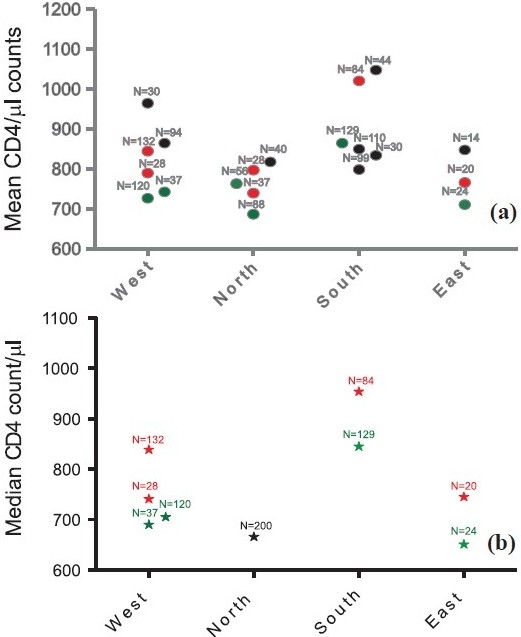
Comparison of mean and median absolute CD4 counts in normal healthy individuals as reported from different parts of India: (a) Comparison of mean CD4 counts, (b) comparison of median CD4 counts from different regions of India. Mean/median CD4 counts in cells/µl is plotted on Y axis and studies done in different regions of India are plotted on Xaxis. Red and green colours indicate CD4 values from females and males respectively. The black colours indicate CD4 values irrespective of the gender. The number above each value indicates number of samples tested to get the value in the study. *Source of data*: Refs. 18, 20, 36, 61, 63-71, 73-76.

Studies on reference ranges in a multiracial Asian population suggest that there may be a true genetic difference in lymphocyte subsets^54^. Also, the comparison of published data from India and other parts of the world shows that the CD4+ T cell counts differ with the population, suggesting that each population should have its own reference range for lymphocyte subsets. In another study the reference ranges of CD4+ T lymphocyte subsets obtained in an Afro-Caribbean population were compared with those published by the WHO and CDC and no significant differences were observed in any of the lymphocyte subsets indicating that the available information can still be used in that population^74^.

## CD4+T cell counts in HIV infected individuals in India

An Indian study showed that the majority of the HIV infected individuals with CD4 counts of 200-350 cells/µl had higher viral load than that suggested by the International AIDS society^75^ and a cut-off CD4+ T cell count of 243 cells/µl reported in this study distinguished asymptomatic (CDC clinical category A) from symptomatic (CDC clinical category B) individuals^76^. This observation highlights the need of validation of the western cut-off values for the target population. Recently a study conducted by Kitahata *et al*^77^ suggested that the early initiation of the treatment might be important for better prognosis of the HIV infection. The study showed that among patients with a 351-500 CD4+ count, the deferral of antiretroviral therapy was associated with an increase in the risk of death of 69 per cent, as compared with the early initiation of therapy. In the light of this finding, the reference ranges might be of great importance in making decisions on the initiation of the ART.

Decision on initiation of ART or prophylaxis for opportunistic infections (OIs) is a critical issue in the management of HIV infected persons. It has been observed that most of the OIs like cryptosporidiosis, toxoplasmosis, herpes zoster, cryptococcal meningitis, *Pneumocystis jerovici* pneumonia, penicillinosis and CMV retinitis were seen in patients having CD4+ T cells <200 cells/µl. On the contrary, tuberculosis and candidiasis may be seen below the count of 400 cells/µl as observed in one of the Indian studies^71^. This study showed that all patients with CD4+ T cell counts <200 cells/µl were symptomatic. Of the patients with CD4+ T cells count of 201-300 cells/µl, 70 per cent males and 30 per cent females were symptomatic. The presence of oral candidiasis and weight loss were highly predictive of low CD4 counts as reported by Ghate *et al*^78^ and these can be considered as markers of HIV disease progression. A study conducted in south Indian population^79^ reported that tuberculosis is more common in patients with CD4 counts <300 cells/µl, however, it can occur over a wide range of CD4 counts, which may be indirectly influenced by wider range of basal pre-infection CD4 counts in a population indicating importance of information on CD4 counts in healthy individuals.

## Conclusion

The available published data on CD4+ T cell counts in normal healthy Indian adults are not adequate for determining reference ranges for Indian population as these have a number of limitations. The data have been generated using different equipment (flowcytometers, modified flowcytometers and non-conventional methodologies like bead-based assays and ELISA) and methodologies (dual platform or single platform or non conventional methods). Differences in the procedures for sample collection, time of sample collection, methodology used for sample processing (e.g. red cell removal techniques, staining, washing, and fixation), variation in the antibody panel used and analysis including gating strategies make it impossible to pool the data from different studies. Many of the studies had inadequate sample size and hence, the results from these studies cannot be generalized. Only one study conducted by ICMR presented data with adequate sample size that included populations from different parts of the country. However, the single platform technology was not available at the time of study and thus the reference ranges were determined only for percentages and not for the absolute counts. Since absolute CD4+ T cell counts are used for HIV disease monitoring and initiation of ART, it is important to determine the reference values for absolute CD4+ T cell counts in Indian population. Also, the statistical values reported by the studies vary with many of the studies reporting mean instead of median values. Since median values need to be taken into consideration while analyzing the data as varied as CD4 cell counts, the comparison of data reported by different studies is difficult.

In conclusion, the data available on reference ranges for CD4+ T cell counts in Indian population are not comparable and there is a need to generate reference ranges in normal population in a well designed multicentric study. The availability of standardized procedures, pretested and calibrated controls and external quality assurance programmes can help generate robust reference values. The establishment of such reference ranges for CD4+ T cells count would provide region-wise differences, if any, in CD4 counts in India and would serve as baseline data for comparison of studies done for analysis of CD4 counts in HIV infected patients. The CD4 ranges would also help in the management of immunodeficiencies other than HIV and in the assessment of immune reconstitution.

## References

[1] Zhu J, Paul WE (2008). CD4 T cells: fates, functions, and faults. *Blood*.

[2] Mosmann TR, Coffman RL (1989). TH1 and TH2 cells: different patterns of lymphokine secretion lead to different functional properties. *Annu Rev Immunol*.

[3] Pattanapanyasat K, Thakar MR (2005). CD4+ T cell count as a tool to monitor HIV progression & anti-retroviral therapy. *Indian J Med Res*.

[4] (1997). [No authors listed]. 1997 revised guidelines for performing CD4+ T-cell determinations in persons infected with human immunodeficiency virus (HIV). Centers for Disease Control and Prevention. *MMW Recomm Rep*.

[5] Jain A, Atkinson TP, Lipsky PE, Slater JE, Nelson DL, Strober W (1999). Defects of T-cell effector function and post-thymic maturation in X-linked hyper-IgM syndrome. *J Clin Invest*.

[6] Nicholson JK (1989). Use of flow cytometry in the evaluation and diagnosis of primary and secondary immunodeficiency diseases. *Arch Pathol Lab Med*.

[7] Bleesing JJ, Straus SE, Fleisher TA (2000). Autoimmune lymphoproliferative syndrome. A human disorder of abnormal lymphocyte survival. *Pediatr Clin North Am*.

[8] Storek J, Witherspoon RP, Atkinson K (2000). Immunologic reconstitution after haematopoietic stem cell transplantation. *Clinical bone marrow and blood stem cell transplantation*.

[9] Eckschlager T, Prusa R, Hladikova M, Radvanska J, Slaby K, Radvansky J (2004). Lymphocyte subpopulations and immunoglobulin levels in Hodgkin’s disease survivors. *Neoplasma*.

[10] Eckschlager T, Radvanska J, Slabý K, Prusa R, Hochova I, Radvanský J (2009). Changes of blood count, lymphocyte subpopulations and immunoglobulin levels in nephroblastoma long term survivors. *Neoplasma*.

[11] Ducloux D, Carron PL, Motte G, Ab A, Rebibou JM, Bresson-Vautrin C (2002). Lymphocyte subsets and assessment of cancer risk in renal transplant recipients. *Transpl Int*.

[12] Fahey JL, Taylor JM, Detels R, Hofmann B, Melmed R, Nishanian P (1990). The prognostic value of cellular and serologic markers in infection with human immunodeficiency virus type 1. *N Engl J Med*.

[13] Stein DS, Korvick JA, Vermund SH (1992). CD4+ lymphocyte cell enumeration for prediction of clinical course of human immunodeficiency virus disease: a review. *J Infect Dis*.

[14] Lee BW, Yap HK, Chew FT, Quah TC, Prabhakaran K, Chan GS (1996). Age- and sex-related changes in lymphocyte subpopulations of healthy Asian subjects: from birth to adulthood. *Cytometry*.

[15] O’Gorman MR, Gelman R (1997). Inter- and intrainstitutional evaluation of automated volumetric capillary cytometry for the quantitation of CD4- and CD8-positive T lymphocytes in the peripheral blood of persons infected with human immunodeficiency virus. Site Investigators and the NIAID New CD4 Technologies Focus Group. *Clin Diagn Lab Immunol*.

[16] Maini MK, Gilson RJ, Chavda N, Gill S, Fakoya A, Ross EJ (1996). Reference ranges and sources of variability of CD4 counts in HIV-seronegative women and men. *Genitourin Med*.

[17] Miyawaki T, Taga K, Nagaoki T, Seki H, Suzuki Y, Taniguchi N (1984). Circadian changes of T lymphocyte subsets in human peripheral blood. *Clin Exp Immunol*.

[18] Nag VL, Agarwal P, Venkatesh V, Rastogi P, Tandon R, Agrawal SK (2002). A pilot study on observations on CD4 & CD8 counts in healthy HIV seronegative individuals. *Indian J Med Res*.

[19] Tollerud DJ, Clark JW, Brown LM, Neuland CY, Pankiw-Trost LK, Blattner WA (1989). The influence of age, race, and gender on peripheral blood mononuclear-cell subsets in healthy nonsmokers. *J Clin Immunol*.

[20] Uppal SS, Verma S, Dhot PS (2003). Normal values of CD4 and CD8 lymphocyte subsets in healthy indian adults and the effects of sex, age, ethnicity, and smoking. *Cytometry B Clin Cytom*.

[21] Gelman R, Cheng SC, Kidd P, Waxdal M, Kagan J (1993). Assessment of the effects of instrumentation, monoclonal antibody, and fluorochrome on flow cytometric immunophenotyping: a report based on 2 years of the NIAID DAIDS flow cytometry quality assessment program. *Clin Immunol Immunopathol*.

[22] Giorgi JV, Cheng HL, Margolick JB, Bauer KD, Ferbas J, Waxdal M (1990). Quality control in the flow cytometric measurement of T-lymphocyte subsets: the multicenter AIDS cohort study experience. The Multicenter AIDS Cohort Study Group. *Clin Immunol Immunopathol*.

[23] Gratama JW, Kraan J, Van den Beemd R, Hooibrink B, Van Bockstaele DR, Hooijkaas H (1997). Analysis of variation in results of flow cytometric lymphocyte immunophenotyping in a multicenter study. *Cytometry*.

[24] Homburger HA, Rosenstock W, Paxton H, Paton ML, Landay AL (1993). Assessment of interlaboratory variability of immunophenotyping. Results of the College of American Pathologists Flow Cytometry Survey. *Ann N Y Acad Sci*.

[25] Parker JW, Adelsberg B, Azen SP, Boone D, Fletcher MA, Gjerset GF (1990). Leukocyte immunophenotyping by flow cytometry in a multisite study: standardization, quality control, and normal values in the Transfusion Safety Study. The Transfusion Safety Study Group. *Clin Immunol Immunopathol*.

[26] Gratama JW, Kraan J, Keeney M, Granger V, Barnett D (2002). Reduction of variation in T-cell subset enumeration among 55 laboratories using single-platform, three or four-color flow cytometry based on CD45 and SSC-based gating of lymphocytes. *Cytometry*.

[27] World Health Organization, Regional Office for South East Asia, New Delhi. *Laboratory guidelines for enumerating for CD4T cell lymphocytes in the context of HIV/AIDS*.

[28] Mandy FF, Nicholson Janet KA, McDougal Steven J (2003). Guidelines for performing single-platform absolute CD4 + T-cell determinations with CD45 gating for persons infected with human immunodeficiency virus. *Morb Mortal Wkly Rep*, Centers for Disease Control and Prevention, January 31.

[29] Malone JL, Simms TE, Gray GC, Wagner KF, Burge JR, Burke DS (1990). Sources of variability in repeated T-helper lymphocyte counts from human immunodeficiency virus type 1-infected patients: total lymphocyte count fluctuations and diurnal cycle are important. *J Acquir Immune Defic Syndr*.

[30] Carmichael KF, Abayomi A (2006). Analysis of diurnal variation of lymphocyte subsets in healthy subjects in the Caribbean, and its implication in HIV monitoring and treatment. *Afr J Med Med Sci*.

[31] Mientjes GH, van Ameijden EJ, Roos MT, de Leeuw NA, van den Hoek JA, Coutinho RA (1992). Large diurnal variation in CD4 cell count and T-cell function among drug users: implications for clinical practice and epidemiological studies. *AIDS*.

[32] Campbell PJ, Aurelius S, Blowes G, Harvey D (1997). Decrease in CD4 lymphocyte counts with rest; implications for the monitoring of HIV infection. *Int J STD AIDS*.

[33] Hulgan T, Shepherd BE, Raffanti SP, Fusco JS, Beckerman R, Barkanic G (2007). Absolute count and percentage of CD4+ lymphocytes are independent predictors of disease progression in HIV-infected persons initiating highly active antiretroviral therapy. *J Infect Dis*.

[34] Moore DM, Hogg RS, Yip B, Craib K, Wood E, Montaner JS (2006). CD4 percentage is an independent predictor of survival in patients starting antiretroviral therapy with absolute CD4 cell counts between 200 and 350 cells/microL. *HIV Med*.

[35] Gallant JE, Hoffmann C CD4 cell count.

[36] Dayama A, Pandit D, Mudaliar S, Bharadwaj R, Bharucha KE, Shrotri AN (2003). A pilot study on CD4 & CD8 cell counts in healthy HIV seronegative pregnant women. *Indian J Med Res*.

[37] Kotylo PK, Fineberg NS, Freeman KS, Redmond NL, Charland C (1993). Reference ranges for lymphocyte subsets in pediatric patients. *Am J Clin Pathol*.

[38] Embree J, Bwayo J, Nagelkerke N, Njenga S, Nyange P, Ndinya-Achola J (2001). Lymphocyte subsets in human immunodeficiency virus type 1-infected and uninfected children in Nairobi. *Pediatr Infect Dis J*.

[39] Ikinciogullari A, Kendirli T, Dogu F, Egin Y, Reisli I, Cin S (2004). Peripheral blood lymphocyte subsets in healthy Turkish children. *Turk J Pediatr*.

[40] Lugada ES, Mermin J, Kaharuza F, Ulvestad E, Were W, Langeland N (2004). Population-based hematologic and immunologic reference values for a healthy Ugandan population. *Clin Diagn Lab Immunol*.

[41] Kiepiela P, Coovadia HM, Coward P, Woodhead R, Abdool-Karim SS, Becker P (1989). Age-related lymphocyte sub-population changes among healthy Africans from birth to adulthood. *Ann Trop Paediatr*.

[42] Tugume SB, Piwowar EM, Lutalo T, Mugyenyi PN, Grant RM, Mangeni FW (1995). Hematological reference ranges among healthy Ugandans. *Clin Diagn Lab Immunol*.

[43] Lin SC, Chou CC, Tsai MJ, Wu KH, Huang MT, Wang LH (1998). Age-related changes in blood lymphocyte subsets of Chinese children. *Pediatr Allergy Immunol*.

[44] O’Gorman MR, Zijenah LS (2008). CD4 T cell measurements in the management of antiretroviral therapy - A review with an emphasis on pediatric HIV-infected patients. *Cytometry B Clin Cytom*.

[45] Shahabuddin S, al Ayed IH, el-Rad MO, Qureshi MI (1998). Lymphocyte subset reference ranges in healthy Saudi Arabian children. *Pediatr Allergy Immunol*.

[46] Swaminathan S, Hanna LE, Raja A, Sankaran K, Kumar AN (2003). Age-related changes in blood lymphocyte subsets of south Indian children. *Natl Med J India*.

[47] Bofill M, Janossy G, Lee CA, MacDonald-Burns D, Phillips AN, Sabin C (1992). Laboratory control values for CD4 and CD8 T lymphocytes.Implication s for HIV-1 diagnosis. *Clin Exp Immunol*.

[48] Reichert T, DeBruyere M, Deneys V, Totterman T, Lydyard P, Yuksel F (1991). Lymphocyte subset reference ranges in adult Caucasians. *Clin Immunol Immunopathol*.

[49] Royce RA, Winkelstein W (1990). HIV infection, cigarette smoking and CD4+ T-lymphocyte counts: preliminary results from the San Francisco Men’s Health Study. *AIDS*.

[50] Vithayasai V, Sirisanthana T, Sakonwasun C, Suvanpiyasiri C (1997). Flow cytometric analysis of T-lymphocytes subsets in adult Thais. *Asian Pac J Allergy Immunol*.

[51] Kalinkovich A, Weisman Z, Burstein R, Bentwich Z (1998). Standard values of T-lymphocyte subsets in Africa. *J Acquir Immune Defic Syndr Hum Retrovirol*.

[52] Jiang W, Kang L, Lu HZ, Pan X, Lin Q, Pan Q (2004). Normal values for CD4 and CD8 lymphocyte subsets in healthy Chinese adults from Shanghai. *Clin Diagn Lab Immunol*.

[53] Al Qouzi A, Al Salamah A, Al Rasheed R, Al Musalam A, Al Khairy K, Kheir O (2002). Immunophenotyping of peripheral blood lymphocytes in Saudi men. *Clin Diagn Lab Immunol*.

[54] Chng WJ, Tan GB, Kuperan P (2004). Establishment of adult peripheral blood lymphocyte subset reference range for an Asian population by single-platform flow cytometry: influence of age, sex, and race and comparison with other published studies. *Clin Diagn Lab Immunol*.

[55] Kam KM, Leung WL, Kwok MY, Hung MY, Lee SS, Mak WP (1996). Lymphocyte subpopulation reference ranges for monitoring human immunodeficiency virus-infected Chinese adults. *Clin Diagn Lab Immunol*.

[56] Aina O, Dadik J, Charurat M, Amangaman P, Gurumdi S, Mang E (2005). Reference values of CD4 T lymphocytes in human immunodeficiency virus-negative adult Nigerians. *Clin Diagn Lab Immunol*.

[57] Yaman A, Cetiner S, Kibar F, Tasova Y, Seydaoglu G, Dundar IH (2005). Reference ranges of lymphocyte subsets of healthy adults in Turkey. *Med Princ Pract*.

[58] Tsegaye A, Messele T, Tilahun T, Hailu E, Sahlu T, Doorly R (1999). Immunohematological reference ranges for adult Ethiopians. *Clin Diagn Lab Immunol*.

[59] Ngowi BJ, Mfinanga SG, Bruun JN, Morkve O (2009). Immunohaematological reference values in human immunodeficiency virus-negative adolescent and adults in rural northern Tanzania. *BMC Infect Dis*.

[60] Bussmann H, Wester CW, Masupu KV, Peter T, Gaolekwe SM, Kim S (2004). Low CD4+ T-lymphocyte values in human immunodeficiency virus-negative adults in Botswana. *Clin Diagn Lab Immunol*.

[61] Saxena RK, Choudhry V, Nath I, Das SN, Paranjape RS, Babu G (2004). Normal ranges of some select lymphocyte subpopulations in peripheral blood of normal healthy Indians. *Curr Sci*.

[62] Kannangai R, Prakash KJ, Ramalingam S, Abraham OC, Mathews KP, Jesudason MV (2000). Peripheral CD4+/CD8+ T-lymphocyte counts estimated by an immunocapture method in the normal healthy south Indian adults and HIV seropositive individuals. *J Clin Virol*.

[63] Murugavel KG, Balakrishnan P, Mohanakrishnan J, Solomon SS, Shankar EM, Muthu Sundaram SP (2009). Establishment of T-lymphocyte subset reference intervals in a healthy adult population in Chennai, India. *Indian J Med Res*.

[64] Amatya R, Vajpayee M, Kaushik S, Kanswal S, Pandey RM, Seth P (2004). Lymphocyte immunophenotype reference ranges in healthy Indian adults: implications for management of HIV/AIDS in India. *Clin Immunol*.

[65] Ray K, Gupta SM, Bala M, Muralidhar S, Kumar J (2006). CD4/CD8 lymphocyte counts in healthy, HIV-positive individuals & AIDS patients. *Indian J Med Res*.

[66] Attili VS, Sundar S, Singh VP, Rai M (2005). Validity of existing CD4+ classification in north Indians, in predicting immune status. *J Infect*.

[67] Ramalingam S, Kannangai R, Zachariah A, Mathai D, Abraham C (2001). CD4 counts of normal and HIV-infected south Indian adults: do we need a new staging system?. *Natl Med J India*.

[68] Shahapur PR, Bairy I, Shivananda PG (2008). CD4 and CD8 reference counts in normal healthy south-Indian adults. *Indian J Med Microbiol*.

[69] Das BR, Bhanushali AA, Khadapkar R, Jeswani KD, Bhavsar M, Dasgupta A (2008). Reference ranges for lymphocyte subsets in adults from western India: Influence of sex, age and method of enumeration. *Indian J Med Sci*.

[70] Singh YG, Dar L, Singh NG (2000). Levels of CD4 and CD8 among the inhabitants of Manipur, India. *J Commun Dis*.

[71] Singh HR, Singh NG, Singh TB (2007). Estimation of CD4+ and CD8+ T-lymphocytes in human immunodeficiency virus infection and acquired immunodeficiency syndrome patients in Manipur. *Indian J Med Microbiol*.

[72] Lifson JD, Finch SL, Sasaki DT, Engleman EG (1985). Variables affecting T-lymphocyte subsets in a volunteer blood donor population. *Clin Immunol Immunopathol*.

[73] Rungta A, Hooja S, Vyas N, Rishi S, Rao A, Gupta S (2008). Enumeration of CD4 and CD8 T lymphocytes in healthy HIV seronegative adults of northwest India: a preliminary study. *Indian J Pathol Microbiol*.

[74] Branch S, Broome H, Abayomi A (2006). Characteristics of lymphocyte subsets in a normal Afro-Caribbean population and the implications in HIV management. *Afr J Med Sci*.

[75] Hammer SM, Saag MS, Schechter M, Montaner Julio SG, Schooley RT, Jacobse DM (2006). Treatment for adult HIV infection: 2006 Recommendations of the International AIDS Society-USA Panel. *JAMA*.

[76] Kannangai R, Kandathil AJ, Ebenezer DL, Nithyanandam G, Samuel P, Abraham OC (2008). Evidence for lower CD4+ T cell and higher viral load in asymptomatic HIV-1 infected individuals of India: implications for therapy initiation. *Indian J Med Microbiol*.

[77] Kitahata MM, Gange SJ, Abraham AG, Merriman B, Saag MS, Justice AC (2009). Effect of early versus deferred antiretroviral therapy for HIV on survival. *N Engl J Med*.

[78] Ghate MV, Mehendale SM, Mahajan BA, Yadav R, Brahme RG, Divekar AD (2000). Relationship between clinical conditions and CD4 counts in HIV-infected persons in Pune, Maharashtra, India. *Natl Med J India*.

[79] Kumarasamy N, Solomon S, Flanigan TP, Hemalatha R, Thyagarajan SP, Mayer KH (2003). Natural history of human immunodeficiency virus disease in southern India. *Clin Infect Dis*.

